# Corrosion Fatigue of a Nickel-Based Superalloy Disc Rotor with Salt in Air and Sulphur Dioxide Environments

**DOI:** 10.3390/ma18163819

**Published:** 2025-08-14

**Authors:** Yong Li, Helen Davies, Mark Hardy, Catherine Jackson, Mark Whittaker

**Affiliations:** 1 Institute of Structural Materials, Swansea University, Wales SA1 8EN, UK; h.m.davies@swansea.ac.uk (H.D.); m.t.whittaker@swansea.ac.uk (M.W.); 2 Rolls–Royce PLC, P.O. Box 31, Derby DE24 8BJ, UK; mark.hardy@rolls-royce.com (M.H.); catherine.jackson@rolls-royce.com (C.J.)

**Keywords:** corrosion fatigue, nickel superalloy, fatigue strength, low-temperature hot corrosion

## Abstract

The fatigue performance of a recently developed nickel superalloy disc was investigated at 700 °C in two environments: air and sulphur dioxide (SO_2_). Prior to testing, specimens were coated with various amounts of sodium sulphate (Na_2_SO_4_) and sodium chloride (NaCl) mixed salt (98% Na_2_SO_4_ + 2% NaCl), and the influence of both environment and salt loading on corrosion fatigue were assessed. Preliminary results showed that salt exposure in air reduced fatigue strength, with greater damage at higher salt levels. In the SO_2_ environment, fatigue strength dropped even more due to low-temperature hot corrosion (LTHC). The details of the corrosion morphology formed in both air and SO_2_ environments are discussed.

## 1. Introduction

In modern aero-jet engines, the service temperature of gas turbine discs approaches 700 °C, as contaminants such as sodium sulphate (Na_2_SO_4_), sodium chloride (NaCl), etc., are ingested by the engine, so the occurrence of low-temperature hot corrosion (LTHC) is inevitable. A gas-phase-induced acidic fluxing mechanism is now recognized as the principal cause of low-temperature hot corrosion [1,2,3]. In this mechanism, a sulphur trioxide (SO_3_)-bearing gas acidifies the otherwise alkaline molten salts. The resulting acidic salts react with the protective oxide scale (Equation (1)), breaking it down. In the equation, *MO* and *MSO*_4_ represent metal oxides and metal sulphates, respectively. To sustain LTHC, both a minimum SO_3_ partial pressure of *p* = ~10^−6^–10^−4^ atm and a continuous supply of salt are required [2].

(1) $$MO+{SO}_{2}+1/2O_{2}\to MSO_{4}$$

It is well established that the sustained reaction of LTHC requires a continuous supply of SO_2_ [1,2,3]. In a laboratory setting, a gaseous chamber is typically required to maintain a SO_2_/SO_3_ atmosphere to facilitate LTHC and generate characteristic corrosion pit morphologies. Several researchers have performed hot corrosion fatigue tests under SO_2_ environments to study the influence of LTHC on the fatigue behaviour of various nickel-based superalloys [4,5,6]. For instance, D.J. Child et al. [4] reported that the fatigue life of RR1000 alloy was significantly reduced when tested in an air–SO_x_ environment with salt at 700 °C, compared to air-only conditions. Similarly, M.L. Hendery [5] investigated the effect of salt composition on the stress-free and corrosion fatigue behaviour of fine-grained RR1000 at 600 °C, finding that specific salt chemistries led to more aggressive attack and decreased fatigue performance. Y. Li et al. [6] explored the effect of shot peening on a recently developed nickel-based superalloy exposed to air–SO_x_ with a salt coating at 700 °C, noting a significant degradation in fatigue resistance.

Apart from experiments conducted under continuous gas-phase SO_2_ exposure, several research groups have studied the impact of LTHC on low-cycle fatigue (LCF) using salt-coated specimens tested in air. Whitlow et al. [7] examined Udimet 720 alloys at 732 °C and found that the presence of salt severely degraded the LCF life. Likewise, Mahobia et al. [8,9] conducted LCF tests on IN718 specimens coated with salt at 550 °C and 650 °C. Li [10] explored the LCF of FGH96 at 700 °C.

Various researchers have also examined the LCF performance of pre-corroded specimens. Corrosion pits were induced by exposing salt-coated specimens to elevated temperature in air prior to LCF air testing. Gabb and Telesman [11,12] demonstrated that pre-corrosion of the powder metallurgy superalloy ME3 led to a 60–98% reduction in LCF life. Additionally, Jiang [13] investigated the hot corrosion fatigue performance of GH4169 alloy, further confirming the detrimental impact of pre-existing pits on fatigue endurance.

While previous studies have investigated salt-induced fatigue degradation, direct comparisons under identical salt loading in both air and SO_2_ environments are limited. This study addresses this gap by performing comparative fatigue tests on salt-coated specimens exposed to air and SO_2_ to evaluate the specific role of SO_2_ in influencing fatigue behaviour. Additionally, the effect of varying salt loads on fatigue performance in air is assessed. Fractographic analysis and damage evaluation are employed to examine corrosion product formation and the progression from pitting to cracking under cyclic loading.

## 2. Experimental Methods

### 2.1. Materials and Specimen Design

The material under investigation was a recently developed nickel superalloy (Alloy1). This new superalloy offers several advantages over existing alloys, such as enhanced dwell crack growth resistance at temperatures between 700 and 775 °C, greater resistance to creep strain accumulation at 650–800 °C, improved oxidation and hot corrosion resistance at 600–800 °C, etc. [14]. Details of the materials, microstructure, and procession route are reported in [6], and the alloy’s chemical composition is listed in Table 1.

The specimen was a cylindrical bar of Ø4.5 mm and 12 mm gauge length. Surfaces were machined to Ra < 0.25 µm and were subsequently shot peened to achieve a surface condition of 110H intensity, 4A media, and 125% coverage.

### 2.2. Salt Deposition Process

All specimens were degreased in an ultrasonic bath using acetone, followed by a wash with ethanol before salt application. Each specimen was pre-heated to T = ~70 °C and placed onto a turntable. An automated spray system was employed to deposit an aqueous solution containing 98% Na_2_SO_4_ and 2% NaCl onto the specimen’s gauge surface. Rotation facilitated uniform coating distribution, while spray parameters—such as flow rate, nozzle distance, and spray duration—were optimized to ensure consistent coverage and reproducibility. Two nominal salt loadings were used: 0.13 ± 0.03 mg/cm^2^ and 13 ± 0.2 mg/cm^2^. Each specimen was weighed using a high-resolution electronic balance before and after salt application to ensure that the target salt quantity was accurately applied. However, achieving thick salt layers (13 mg/cm^2^) was particularly challenging, as higher salt deposition levels increased the tendency of salt layer detachment from the specimen surface.

### 2.3. Testing

**Figure 1:**
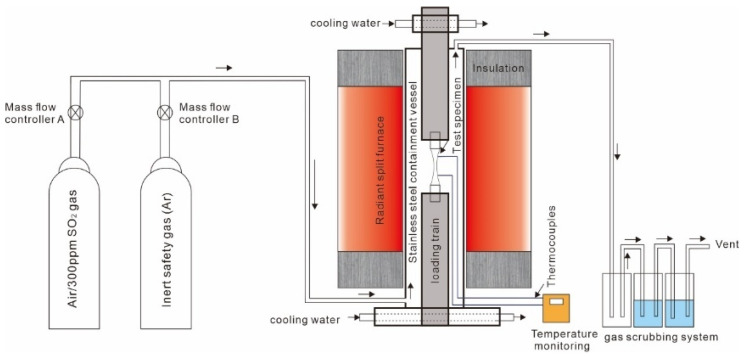
The schematic diagram of the fatigue testing facility in SO_2_ environment [6].

Fatigue tests have been categorized into four groups, according to the salt loading and testing environment, and these are listed in Table 2. All specimens were tested to failure, at 700 °C under various gaseous environments and three stress levels (the normalized stress (based on the highest test stress) is low (0.93), medium (0.97), and high stress (1), respectively). A schematic of the testing system used in the SO_2_ environment is shown in Figure 1. The salt-coated specimen was placed in the load train, with two N-type thermocouples positioned at either end of the specimen’s gauge section to monitor temperature. The chamber was sealed and purged with SO_2_ gas to establish a stable hot corrosion environment at 700 °C, after which the mechanical fatigue test was conducted. The load control tension–compression fatigue tests were conducted on an Instron 8800 (Instron, Norwood, MA, USA) under a triangular waveform with a six second period at a fixed R ratio of −1. In the SO_2_ environment testing, a 300 ppm concentration of SO_2_ gas with a flow rate of 80 cm^3^ per minute was employed. For the Group 2 and 4 tests, each test was interrupted every 50 h, and salt was re-applied to the specimen.

### 2.4. Post-Test Analysis

After failure, the fracture surfaces of all specimens were thoroughly examined to identify the crack initiation sites. Selected specimens were sectioned parallel to the loading direction to assess corrosion damage using a Hitachi 3500 scanning electron microscope (SEM) (Hitachi, Tokyo, Japan) equipped with an Oxford Instruments EDX energy-dispersive X-ray spectroscopy system (Oxford Instrument, Oxford, UK). Surface integrity of each salt-coated specimen was monitored every 50 h using a Nikon camera (Nikon, Tokyo, Japan) and Hitachi 3500 SEM (Hitachi, Tokyo, Japan). The depth of the work hardening layer (WHL) produced by shot peening—both before and after testing under high stress levels—was evaluated using electron backscatter diffraction (EBSD) mapping with the Hitachi 3500 SEM (Hitachi, Tokyo, Japan).

## 3. Results

### 3.1. Surface Observation After 50 h Fatigue Test in Air and in SO_2_

**Figure 2:**
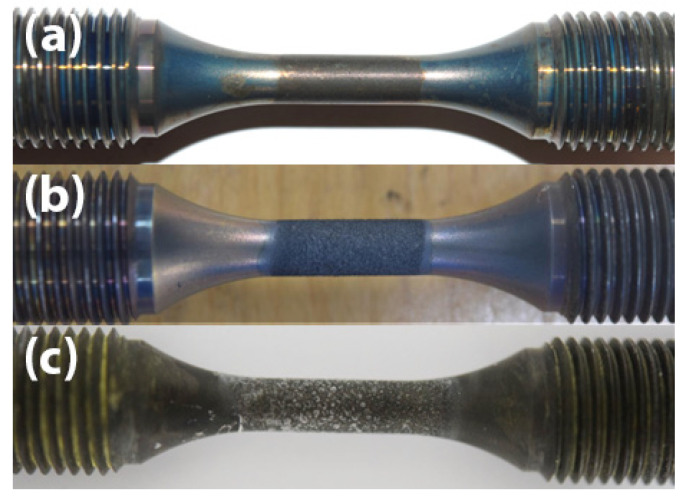
Surface observation after 50 h of fatigue testing in air with 0.13 mg/cm^2^ of salt (**a**), 0.13 mg/cm^2^ of salt in SO_2_ (**b**), and 13 mg/cm^2^ of salt in air (**c**).

Figure 2a,b show the surface appearance of specimens coated with 0.13 mg/cm^2^ salt tested in air and in SO_2_ environments, respectively. Distinct colour differences were apparent on the specimen surfaces within the gauge section.

After 50 h (Figure 2a), the gauge presented a brownish colour with a relatively smooth surface retained in the specimen tested in air, while a black, rough surface with spallation was observed in the specimen tested in SO_2_ (Figure 2). In the thick salt specimen (13 mg/cm^2^) tested in air (Figure 2c), yellowish residual salts were loosely attached to the surface, and no spallation was observed on the specimen surface by eye.

**Figure 3:**
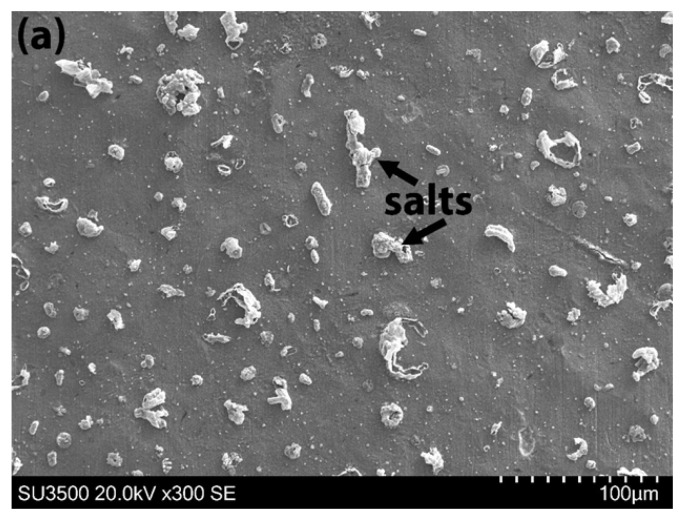

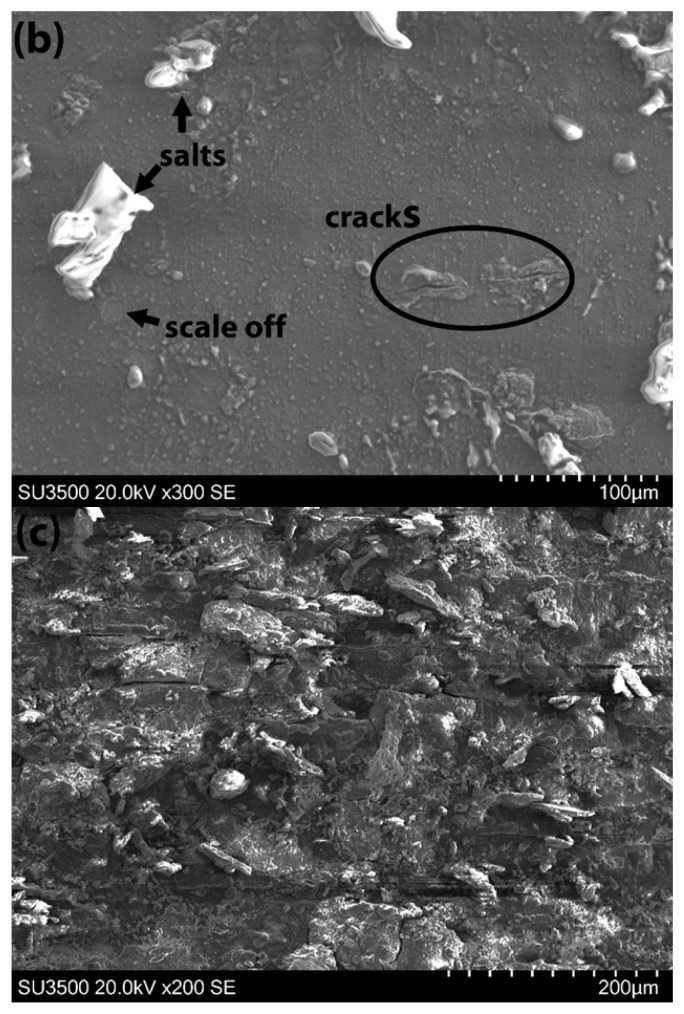
Surface inspection after 50 h of testing at the high stress level by SEM: (**a**) 0.13 mg/cm^2^ specimen tested in air; (**b**) 13 mg/cm^2^ specimen tested in air; (**c**) 0.13 mg/cm^2^ specimen tested in SO_2_.

Detailed surface analysis of the specimens tested in air was conducted using SEM, as shown in Figure 3. Salt particles were observed on the undulated, indented surfaces, where machining marks remained visible. In specimens with a 0.13 mg/cm^2^ salt deposition tested under high stress, occasional shallow surface cracks associated with salt were detected (Figure 3a). In contrast, specimens with a 13 mg/cm^2^ salt deposition exhibited thick residual salt on the surface and a higher frequency of surface micro-cracks. Additionally, patches of thin surface scales appeared to have spalled off (Figure 3b).

A distinct difference in surface appearance was observed between specimens tested in air and those exposed to an SO_2_ environment. No residual salt was present on the surface of the specimen tested in SO_2_. Instead, widespread surface damage was evident, including thick scale formation along the entire gauge length, significant surface spallation, and numerous surface cracks (Figure 3c).

### 3.2. Fatigue Behavior

**Figure 4:**
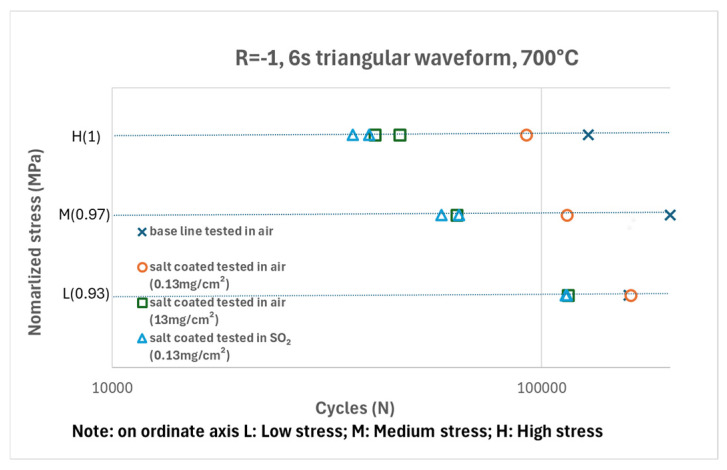
S-N data from specimens tested in air and SO_2_ environment.

Fatigue data from all test conditions are plotted in Figure 4. All tests demonstrated a consistent trend under the corresponding test conditions, except for one baseline specimen tested in air, which exhibited early failure. In general, the fatigue life of all specimens coated with salt decreased in air tests, with the reduction becoming more pronounced as the salt amount increased.

When comparing the datasets for specimens exposed to 13 mg/cm^2^ of salt and tested in air with those exposed to 0.13 mg/cm^2^ of salt and tested in SO_2_, the results are difficult to distinguish at low stress levels. However, at higher stress levels, specimens tested in air showed slightly better performance. Nonetheless, this difference remains within the experimental scatter band.

### 3.3. Fractography

The crack initiation site in each specimen is listed in Table 3.

**Figure 5:**
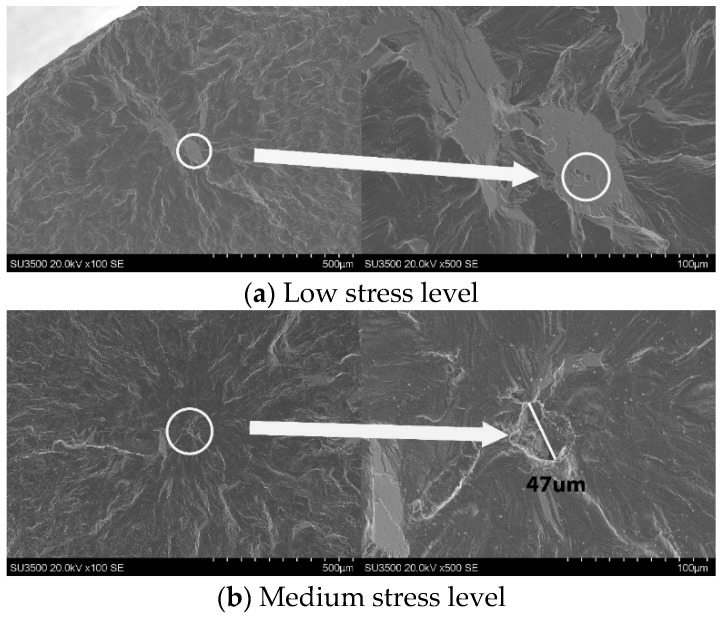

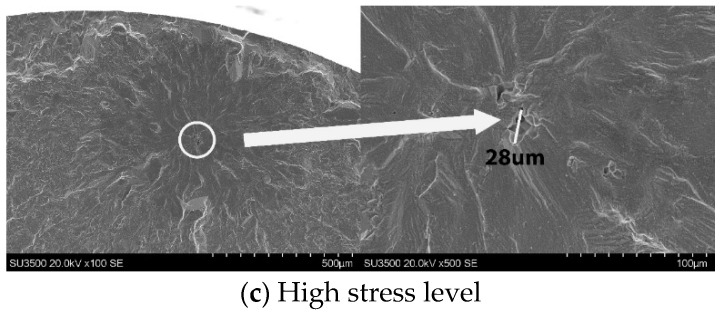
Fractography for baseline specimens tested in air: (**a**) low stress level; (**b**) medium stress level; (**c**) high stress level.

Fractographs of all the baseline specimens tested in air are shown in Figure 5. Invariably, all specimens illustrated single sub-surface crack initiation sites. At the low stress level (Figure 5a), the initiation site featured a large, inclined facet containing micro-pores with a maximum Feret diameter of 90 µm, which were likely responsible for the early failure of the specimen.

In the other two specimens, cracks originated from micro-pores and propagated approximately 200–300 µm before transitioning into a general transgranular fracture mode. Irregularly shaped micro-pores, with maximum Feret diameters of 47 µm and 28 µm, were observed in each specimen (Figure 5b,c).

**Figure 6:**
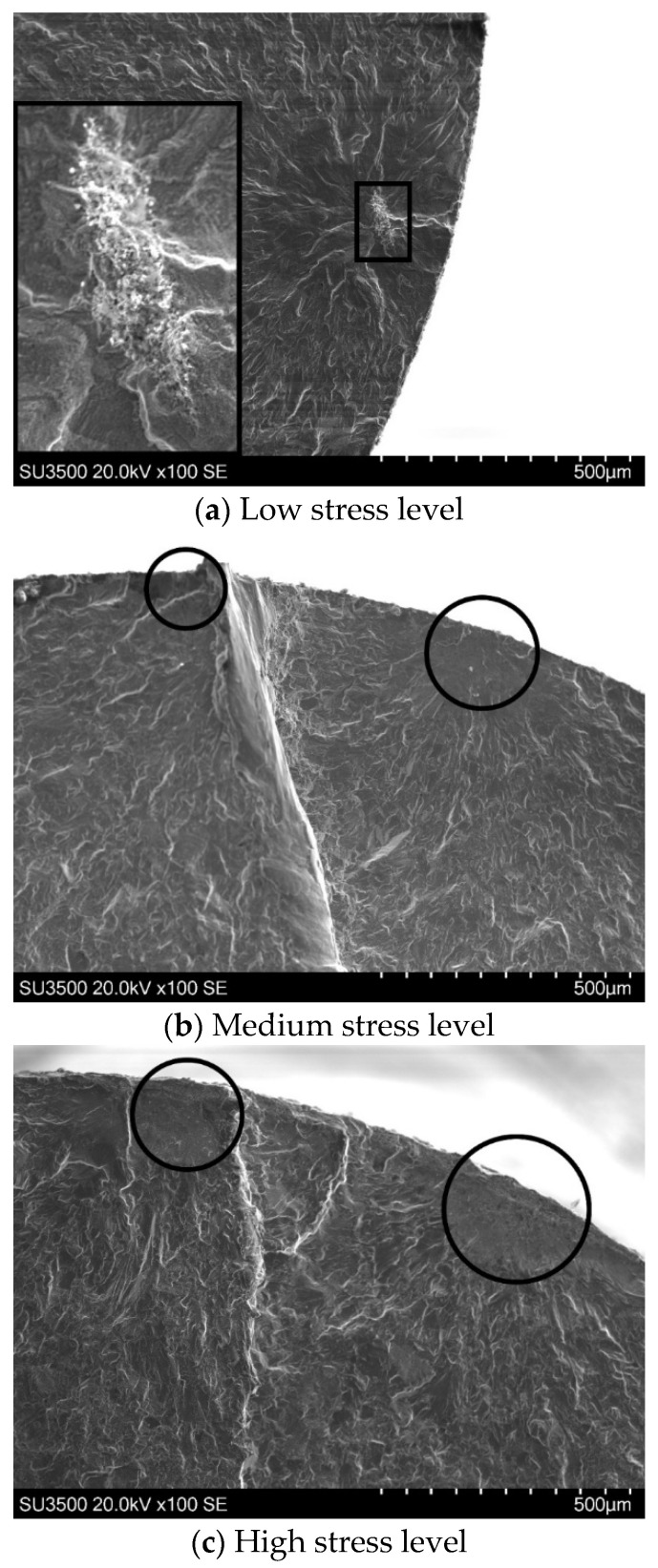
Fracture surfaces of specimens with 0.13 mg/cm^2^ of salt tested in air: (**a**) low stress level; (**b**) medium stress level; (**c**) high stress level.

A mixture of surface and sub-surface failure modes was observed in 0.13 mg/cm^2^ salt-coated specimens tested in air (Figure 6).

At the low stress level, the crack nucleated from a sub-surface site was identified by EDX mapping as an aluminide oxide inclusion (Figure 6a). At the medium stress level, two displaced surface initiation sites were observed on the fracture surface, as circled in Figure 6b. The “rise” in the step formed between adjacent cracks as they coalesced suggests evidence of mechanical shear abrasion. At the high stress level, the fracture surface resembled that of the medium stress level, with multiple surface initiation sites and coalescence steps evident on the final fracture plane (Figure 6c).

**Figure 7:**
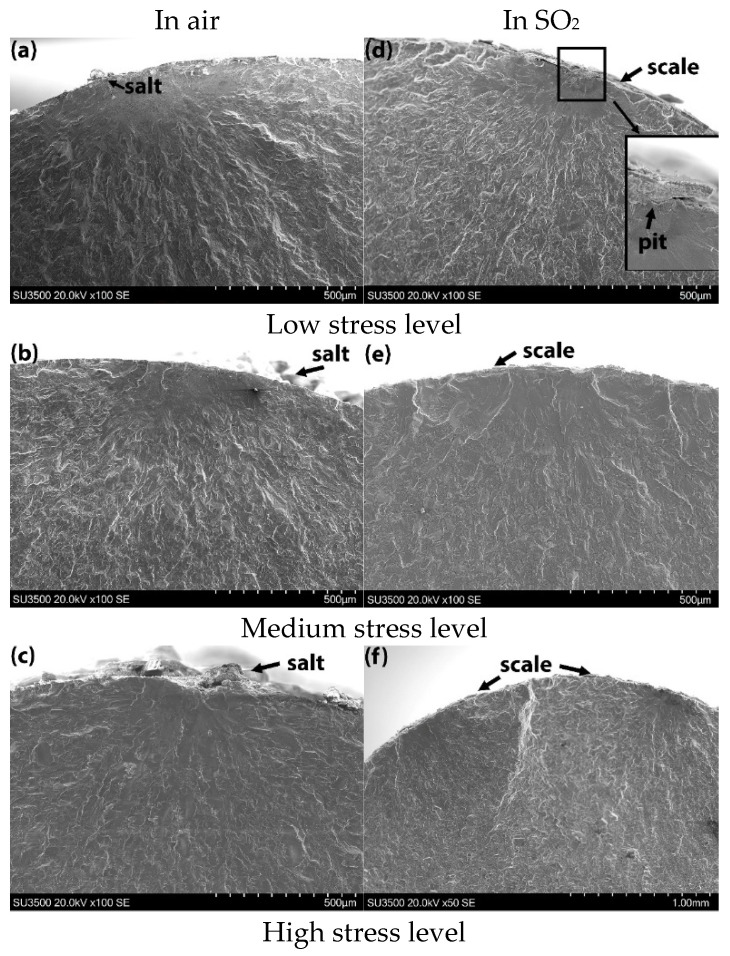
Fractography for specimens with 13 mg/cm^2^ of salt tested in air and 0.13 mg/cm^2^ of salt tested in SO_2_: (**a**,**d**) low stress level; (**b**,**e**) medium stress level; (**c**,**f**) high stress level.

Fracture surfaces of specimens tested in air with 13 mg/cm^2^ of salt and in SO_2_ with 0.13 mg/cm^2^ of salt are shown in Figure 7. All specimens exhibited surface-related crack initiation. In the air test, the specimen edges appeared relatively smooth, and residual salt deposits were observed on the surface. One notable exception was a crater observed on the edge (Figure 7c), suggesting either a corrosion pit or the detachment of a grain. In contrast, specimens tested in the SO_2_ environment showed slightly rougher edges, with a porous scale evident around the specimen perimeter. At the low stress level, crack initiation was traced to a corrosion pit, indicating that pit-induced corrosion was responsible for early-stage cracking. For the medium stress level, a broad crack initiation zone was observed, while at the high stress level, two distinct initiation sites were identified.

### 3.4. Characterization of “Corrosion Damage” in SO_2_ and in Air

A selection of the failed specimens, tested under the high cyclic stress conditions and various salt amounts and gas environments, was metallographically sectioned to investigate the general form of corrosion damage and morphology.

**Figure 8:**
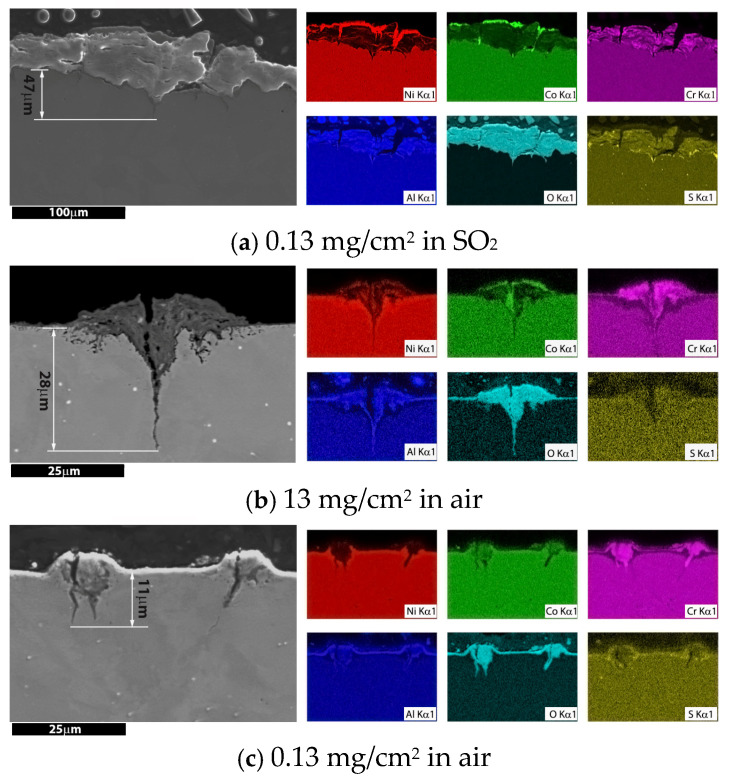
Metallographic section through typical “corrosion damage” and EDX mapping, illustrating damage morphology and chemical element distribution: (**a**) 0.13 mg/cm^2^ of salt in SO_2_; (**b**) 13 mg/cm^2^ of salt in air; (**c**) 0.13 mg/cm^2^ of salt in air.

Typical examples of corrosion and cracking morphologies are shown in Figure 8. Clear differences are observed between specimens tested in air and those exposed to the SO_2_ environment. In the SO_2_ environment, a thick, continuous oxide scale was present across the specimen gauge section, characterized by “V-shaped” damage consisting of a pit and associated cracks. SEM inspection revealed a banded internal structure, while EDX analysis (Figure 8a) identified distinct compositional variations through the thickness of the scale. Nickel and cobalt were detected in the outermost layers, with chromium and aluminium oxides dominating the mid-layers.

At the interface with the substrate, elevated concentrations of chromium and sulphur were found, and a very thin sulphur-rich band was observed at the corrosion product/alloy interface. The depth of the corrosion pit extended to 47 µm.

In the specimen with 13 mg/cm^2^ of salt tested in air, a very thin continuous oxidation layer was observed, occasionally interrupted by crown-like features on the surface. Beneath these crowns, “V-shaped” damage (a combination of pits and cracks) was identified. EDX analysis revealed the composition of these features (Figure 8b). The outermost layer of the crown was rich in cobalt and nickel, followed by a layer of corrosion products composed of chromium and aluminium embedded into the substrate. A nickel-rich band was observed at the interface with the substrate. The depth of the combined corrosion pit and crack was approximately 28 µm.

The corrosion morphology of the 0.13 mg/cm^2^ specimen tested in air was similar to the 13 mg/cm^2^ specimen: a continuous aluminium oxidation layer formed on the surface, small chromium oxide corrosion pits were evidenced under certain salt deposit locations, and the depth of the corrosion feature reached approximate 10 mm. A very thin sulphide was deposited in the interface, as shown in Figure 8c.

**Figure 9:**
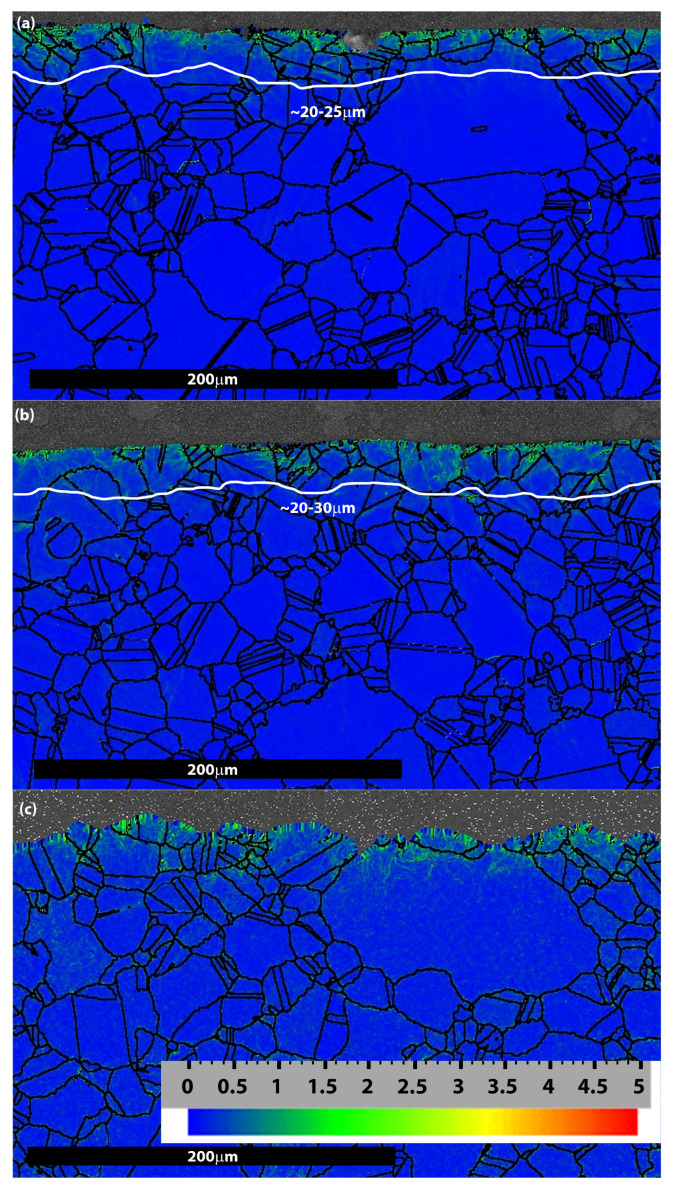
Residual WHL depth after fatigue testing of three different environmental conditions at the high stress level: (**a**) 0.13 mg/cm^2^ of salt in air; (**b**) 13 mg/cm^2^ of salt in air, (**c**) 0.13 mg/cm^2^ of salt in SO_2_.

EBSD maps of the near-surface regions in post-test specimens subjected to high stress levels are shown in Figure 9. Misorientation was indicated by the colour difference, and the low angle range of 0–2° is represented by a blue (0°) and green (2°) colour scale within all EBSD maps. The depth of the original work-hardening layer generated by shot peening was approximately 25–35 µm, as illustrated in [6].

The original work-hardening layer is retained in both specimens tested in air regardless of the salt loading (see Figure 8b and Figure 9a). Conversely, in the specimen tested in SO_2_, a wavy surface was observed, and the whole work-hardening layer almost vanished as significant surface spallation occurred. The colour difference in the map is probably produced by surface cracking and the formation of sulphides, as shown in Figure 9c.

## 4. Discussion

The current testing results clearly confirm the detrimental effect of salt on fatigue performance. The salt-coated specimens exhibited reduced fatigue resistance when tested in air. Fatigue strength showed at least a 30% reduction in the 0.13 mg/cm^2^ salt-coated specimens tested in air (excluding the baseline specimen with early failure). As the salt concentration increased, fatigue resistance decreased further. The introduction of SO_2_ gas into the tests significantly accelerated the degradation of material fatigue performance compared to tests with the same salt loading conducted in air. Notably, the thick salt specimen with 13 mg/cm^2^ tested in air produced similar results to the 0.13 mg/cm^2^ specimen tested in SO_2_.

As is well established, structural fatigue failure can be divided into three stages: crack initiation (nucleation or early damage formation), crack propagation (microstructure-sensitive crack growth and coalescence), and final fracture. Both the initiation and propagation stages can be significantly influenced by the service environment.

It appears that both the crack initiation and early crack propagation stages were significantly affected in the specimen coated with salt and tested in the SO_2_ environment at 700 °C, where low-temperature hot corrosion (LTHC) was promoted. As expected under these conditions, stabilized liquid eutectic melts of Na_2_SO_4_–nickel sulphate (NiSO_4_) (T_m_ = 671 °C) and Na_2_SO_4_–cobalt sulphate (CoSO_4_) (T_m_ = 565 °C) were formed due to the sufficient partial pressure of SO_3_. These molten salts compromised the integrity of the continuous protective oxide scale through a fluxing mechanism at the substrate interface, facilitating corrosion-assisted damage and accelerating crack development.

Simultaneously, the general corrosion scale that forms on the surface diminishes the beneficial effects of shot peening. Shot peening introduces compressive residual stresses and creates a work-hardened surface layer, both of which enhance resistance to crack initiation and delay pit-to-crack transition. However, as illustrated in Figure 9, the repeated exposure to molten salt significantly reduces—or even eliminates—the work-hardened layer.

In contrast, for specimens tested in air, the absence of a significant partial pressure of SO_3_ prevents the stabilization of a liquid Na_2_SO_4_–NiSO_4_ eutectic; thus, typical low-temperature hot corrosion (LTHC) does not occur. Nonetheless, metallographic cross-sections of air-tested specimens revealed localized corrosion features—such as pits and fissures—particularly at sites associated with residual salt deposits (Figure 8). These localized defects serve as favourable sites for fatigue crack initiation.

The following assumptions may help clarify the mechanism behind the formation of corrosion pits. The first assumption involves the formation of a eutectic phase, which attacks the substrate at 700 °C. S. Kameswari et al. [15] performed differential thermal analysis (DTA) on a mixture containing 1% NaCl and Na_2_SO_4_, revealing an endothermic peak between 630 °C and 650 °C, indicative of eutectic formation. Moreover, during corrosion tests on Nimonic alloy 90 [15,16], the most severe corrosion occurred around 750 °C when exposed to the same salt mixture. Consistent with our experimental results (Figure 8), the observed corrosion damage suggests that eutectic salts can form and initiate material corrosion in the binary salt mixture of 98% Na_2_SO_4_ and 2% NaCl used in this study.

Another hypothesis is the localized release of SO_3_ from Na_2_SO_4_, which may generate a high partial pressure of SO_3_ sufficient to initiate low-temperature hot corrosion (LTHC). This effect is likely more pronounced in specimens with higher salt loading, as more SO_3_ is released during decomposition. For instance, cobalt oxide was detected in the corrosion product of the specimen with 13 mg/cm^2^ salt loading tested in air (Figure 8b) but was absent in the 0.13 mg/cm^2^ specimen tested in SO_2_. This observation suggests that the decomposition reaction, Na_2_SO_4_ → sodium oxide (Na_2_O) + SO_3_, proceeds in favour of SO_3_ release under certain local conditions. The liberated SO_3_ can react to form a Na_2_SO_4_–CoSO_4_ eutectic (melting point 565 °C), which subsequently attacks the substrate at 700 °C, resulting in the incorporation of cobalt into the corrosion product.

Although chloride is not the focus of this discussion, previous studies [17,18] have shown that it can play a significant role in modifying corrosion product morphology, particularly under air exposure.

In terms of corrosion fatigue performance, specimens tested in air showed reduced fatigue resistance when coated with salt, largely due to the formation of corrosion pits of varying sizes (Figure 8). These pits act as stress concentrators, increasing local stress and accelerating crack initiation. In the present study, pit growth was primarily governed by the available salt quantity. Once the salt was exhausted, pit development ceased. Consequently, larger pits formed in high-salt-loading specimens—reaching 28 µm in the 13 mg/cm^2^ case—compared to only 10 µm in the 0.13 mg/cm^2^ case. Pit size influenced not only the pit-to-crack transition but also the remaining depth of the work-hardened layer (WHL), which is known to resist crack propagation.

Significant differences in corrosion morphology were observed between air and SO_2_ environments. However, notably, the 13 mg/cm^2^ specimen tested in air exhibited similar fatigue resistance to the 0.13 mg/cm^2^ specimen tested in SO_2_. The early-stage pit morphology likely played a critical role in this outcome. In the SO_2_ environment, the accelerated LTHC process destroyed the continuous protective oxide scale, forming a porous corrosion layer and promoting localized pit formation. At the early LTHC stage, rather than forming deep “V-shaped” pits, the molten salts spread along the surface, creating a broad, wavy corrosion front with many shallow pits. These shallow pits were distributed uniformly across the gauge section. With continued chemical and mechanical degradation, pits grew and coalesced, eventually forming dominant “V-shaped” pits that transitioned into cracks.

In contrast, for high-salt-loading specimens tested in air, corrosion pits tended to form only beneath isolated salt deposits. These localized pits created strong stress concentrations, from which critical cracks could initiate. Previous research on the elastic stress concentration factor (*Kt*) of hot corrosion pits in ME3 alloy reported values ranging from 1.36 to 2.85, with an average around 2.15 [11,12]. For a single pit in René 104 alloy, modelled as a hemispherical pit (depth-to-diameter ratio *d*/*w* = 1), the *Kt* at the pit tip was calculated to be three [19]. Therefore, although the overall corrosion rate was higher in the SO_2_ environment, the average stress concentration factor of the more distributed, shallow pits was lower than that of the deep, isolated pits in air.

Additionally, the reduction in the WHL in the SO_2_-tested specimens accelerated both the pit-to-crack transition and subsequent crack growth. These combined effects—stress concentration, surface integrity, and WHL degradation—collectively governed the observed corrosion fatigue performance.

## 5. Conclusions

Corrosion fatigue performance of a recently developed superalloy disc coated with a 98% Na_2_SO_4_ and 2% NaCl mixed salt has been evaluated at two different surface loadings (0.13 mg/cm^2^ and 13 mg/cm^2^) at 700 °C, in both air and an SO_2_-containing atmosphere. Based on preliminary results, the following conclusions can be drawn:

- Fatigue strength is highly sensitive to both salt concentration and gas environment.
- Fatigue strength dropped up to 30% at 0.13 mg/cm^2^ salt loading in air. Higher salt level resulted in further deduction.
- For a given salt concentration, SO_2_ exposure caused severe degradation via low-temperature hot corrosion.
- Corrosion pits and fissures formed in air, while thick, porous, and non-protective oxide scales together with corrosion pits developed in the SO_2_ environment. The beneficial effects of shot peening were lost in SO_2_ due to surface degradation caused by molten salt attack.

In this study, corrosion pits were observed even in air due to the applied mixed salt. However, the detailed mechanisms responsible for hot corrosion pit formation in air remain unclear, indicating the need for further investigation.

## Figures and Tables

**Table 1:** The composition of the nickel superalloy [6].

wt.%	Ni	Co	Cr	Fe	Mn	Mo	W	Ai	Ti	Ta	Nb	Si	C	B	Zr	Hf
Min.	Bal.	14.60	11.50	0.80	0.20	2.00	3.30	2.90	2.60	3.50	1.20	0.10	0.02	0.01	0.05	0.000
Max.	Bal.	15.90	13.00	1.20	0.60	2.40	3.70	3.30	3.10	5.10	1.80	0.60	0.06	0.03	0.11	0.045

**Table 2:** Details of the salt and testing gas environment.

Group	Surface Condition	Salt Amount (mg/cm^2^)	Gas Environment
1	110H-4A-125%	0	air
2	110H-4A-125%	0.13	air
3	110H-4A-125%	13	air
4	110H-4A-125%	0.13	SO_2_

**Table 3:** Crack initiation site in specimens tested in air.

Salt Amount	Stress Level	Crack Initiation Site
0 (No salt)	Low	Sub-surface
	Medium	Sub-surface
	High	Sub-surface
0.13 mg/cm^2^	Low	Surface
	Medium	Surface
	High	Surface
13 mg/cm^2^	Low	Surface
	Medium	Surface
	High	Surface

## Data Availability

The data presented in this study are available on request from the corresponding author. The data are not publicly available due to commercial confidentiality.
